# Retroperitoneal solitary fibrous tumor: surgery as first line therapy

**DOI:** 10.1186/s13569-015-0034-y

**Published:** 2015-08-27

**Authors:** Rahul Rajeev, Mohit Patel, Thejus T. Jayakrishnan, Fabian M. Johnston, Meena Bedi, John Charlson, Kiran K. Turaga

**Affiliations:** Division of Surgical Oncology, Department of Surgery, Medical College of Wisconsin, 9200 West Wisconsin Avenue, Milwaukee, WI 53226 USA; Department of Radiation Oncology, Medical College of Wisconsin, 8701 Watertown Plank Rd., Milwaukee, WI 53226 USA; Section of Hematology and Oncology, Medical College of Wisconsin, 9200 West Wisconsin Avenue, Milwaukee, WI 53226 USA

**Keywords:** Solitary fibrous tumors, Retroperitoneal sarcoma, Adjuvant chemotherapy, Adjuvant radiotherapy, Surgical outcomes, Surgical Oncology

## Abstract

**Background:**

Solitary fibrous tumors (SFT) of the retroperitoneum are rare spindle cell neoplasms, with a paucity of data on treatment outcomes. We hypothesized that surgical excision offered acceptable outcomes in SFTs.

**Methods:**

The National Cancer Database (NCDB) was used to identify patients with SFT from 2004 to 2011. Primary outcome measures were 30 day mortality and overall survival. Descriptive analyses were performed. Furthermore, a systematic review of published literature was conducted after creating a pre-specified search strategy.

**Results:**

Of 51 patients in the NCDB, 58.8 % (n = 30) were males, with a median age 60 years (IQR 49–72 years). Median tumor size was 16 cm (IQR 11–21 cm). Surgical resection was performed in 92.2 % (n = 47) with 63.8 % (n = 30) having a margin negative resection. Peri-operative mortality was 2.1 % (n = 1). Of survival outcomes available for 18 patients, the median OS was 51.1 months. From the systematic review, we identified 8 studies, with 24 patients. Median age and tumor size was similar to the NCDB [47.5 years (IQR 39–66.5 years), 12 cm (IQR 7–17 cm)]. Majority [91.7 % (n = 22)] underwent surgical excision alone while one received adjuvant chemotherapy and none received radiation. After median follow up of 54 months (IQR 28–144 months), 79.2 % (n = 19) were alive without disease. Three patients (12.5 %) died of disease, one was alive with disease and one was lost to follow up. Recurrence was reported in 16.7 % (n = 4) of patients.

**Conclusion:**

Complete surgical excision is a viable treatment modality for retroperitoneal SFT leading to long term survival. Low recurrence rates would argue against the need for routine adjuvant radiation or chemotherapy.

## Background

Solitary fibrous tumors (SFT) are extremely rare spindle cell neoplasms with a varied presentation. First described as a pleural tumor by Klemperer et al. later they were identified in extra-thoracic sites, most commonly the adrenals, head and neck, retroperitoneum, kidneys, liver and skeletal muscle [1–3]. Existing literature on SFTs are limited to case reports that mostly describe pleural tumors [4, 5]. Owing to its rarity, extra-pleural SFTs have not been studied adequately.

Retroperitoneal SFTs are a distinct sub-group of extra-pleural SFTs, often incidentally discovered during imaging for an unrelated pathology and present with non-specific symptoms, mainly abdominal pain, hip pain and urinary symptoms [6]. Extra-thoracic SFTs have been classically described as benign tumors but recent studies with larger numbers of cases and longer follow up have demonstrated the existence of a subgroup of tumors that exhibit a malignant clinical course with local and distant recurrence even after surgical excision [4, 7, 8]. Adverse outcomes are usually associated with atypical histological features (nuclear pleomorphism, increased cellularity, necrosis or mitoses greater than 4/10 HPF) and size greater than 10 cm [9, 10]. Also, cases of extra-thoracic SFTs with benign features at initial presentation have been reported to recur years after surgical excision with malignant features [9, 11, 12].

Surgical excision has been the standard treatment option for both benign and malignant SFTs but late recurrences have been observed [2, 13]. A multi-modality approach using adjunct radiotherapy and chemotherapy has been utilized in cases where complete excision is not achieved [14]. We hypothesized that surgical excision is an acceptable treatment modality with favorable outcomes for SFTs of retroperitoneal origin.

## Methods

The National Cancer Database (NCDB) was surveyed for cases with a histological diagnosis of solitary fibrous tumors from 2004 to 2011. The NCDB was established in 1989 as a joint project of the American Cancer Society and the Commission on Cancer of the American College of Surgeons. It is a nationwide, facility-based, comprehensive clinical surveillance resource oncology data set that captures 70 % of all newly diagnosed malignancies in the US annually, covering more than 1500 commission-accredited cancer programs (http://www.ncdbpuf.facs.org). Cases diagnosed at autopsy were excluded. Demographic details, tumor characteristics, treatment and follow-up data were abstracted from the database. Primary outcome measures were 30-day mortality and overall survival. Descriptive statistical analysis was performed.

**Fig. 1:**
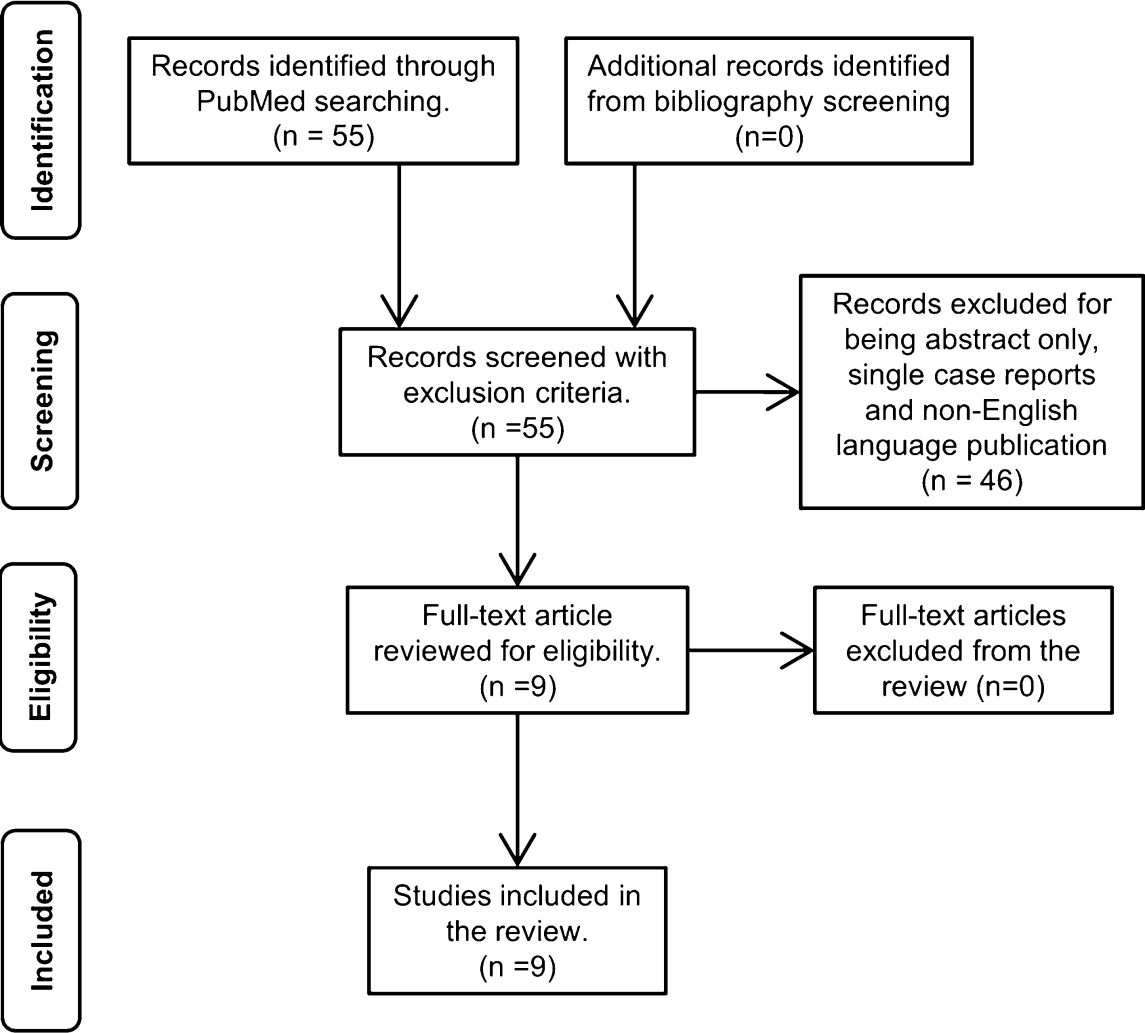
Search strategy for selecting studies for the systematic review

A systematic review of published literature on SFTs was conducted in the MEDLINE database using PubMed using a pre-specified search strategy (Fig. 1). Search terms used were “solitary fibrous tumor” and “retroperitoneum”. Case reports and literature published in non-English languages were excluded from the study. The search strategy returned 55 articles and 8 studies satisfied the selection criteria. Bibliographies of the selected articles were further searched but no additional relevant articles were found. All the selected articles were retrospective cohort studies.

Statistical analysis was performed using Stata Software Version 12.1 (StataCorp, College Station, TX, USA).

## Results

### Patient characteristics

The NCDB database yielded 51 patients with 58.8 % (n = 30) males. Median age was 60 years (IQR 49–72 years). Clinical characteristics of the patients are shown in Table 1.

**Table 1:** Baseline characteristics and therapies of solitary fibrous tumor (SFT) patients from National Cancer Database (NCDB) and systematic review

Characteristic	NCDB (51 pts)		Systematic review (24 pts)	
	Median (IQR)^a^	Frequency N (%)	Median (IQR)^a^	Frequency N (%)
(Age (years)	60 (49–72)		47.5 (39–66.5)	
Gender				
Male		30 (58.8)		14 (58.3)
Female		21 (41.2)		10 (41.7)
Tumor size (cm)	16 (11–21)		12 (7–17)	
<10 cm		12 (23.5)		7 (29.2)
≥10 cm		35 (68.6)		11 (45.8)
Tumor grade				
Well-differentiated		6 (11.76)		
Moderately differentiated		6 (11.76)		
Poorly differentiated		6 (11.76)		
Undifferentiated/anaplastic		5 (9.8)		
Distant metastasis at diagnosis		4 (9.09)		
Surgery		47 (92.2)		22 (91.7)
Surgical margins^b^				
Negative (R0)		30 (63.8)		
Microscopic positive (R1)		2 (4.3)		
Gross positive (R2)		3 (6.4)		
Unknown		12 (25.5)		
Unplanned 30-day readmission^b^		4 (8.5)		
30-day mortality^b^		1 (2.1)		
Radiation therapy		16 (31.4)		
Adjuvant		13 (25.5)		
Neo-adjuvant		3 (5.8)		
Chemotherapy		10 (19.6)		1 (4.2)
Adjuvant		5 (9.8)		
Neo-adjuvant		3 (5.8)		
Systemic		2 (3.9)		
Combination therapy		4 (7.8)		
Late recurrences				4 (16.7)
Follow-up (months)	45.2 (30.3–63.3)		54 (28–144)	
Mortality at last follow-up		10 (19.6)		3 (12.5)

^a^ *IQR* inter-quartile range

^b^Percentages are calculated on the number of patients who had surgery for the tumor

### Tumor characteristics

Median tumor size was 16 cm (IQR 11–21 cm) and 68.6 % of tumors were 10 cm or larger. When separated by tumor grade, 9.8 % (n = 5) were undifferentiated/anaplastic. Well-differentiated, moderately differentiated and poorly differentiated tumors were present in equal proportion of 11.8 % (n = 6). Distant metastases were present at diagnosis in 9.1 % (n = 4) of tumors.

### Surgical treatment

Surgical excision was recorded in 92.2 % (n = 47) of patients. Among 35 patients with recorded surgical margins, microscopically negative (R0) accounted for 63.8 % (n = 30), microscopically positive (R1) in 4.3 % (n = 2) and gross positive (R2) in 6.4 % (n = 3). Peri-operative mortality was 2.1 % (n = 1). Unplanned readmissions within 30 days of surgery were observed in 8.5 % (n = 4) of patients.

### Chemotherapy and radiation therapy

Peri-operative radiation therapy was given to 31.4 % (n = 16) of patients (3 neo-adjuvant, 13 adjuvant). Of the 19.6 % (n = 10) patients who received chemotherapy, 15.6 % (n = 8) were peri-operative (3 neo-adjuvant, 5 adjuvant) and 3.9 % (n = 2) did not undergo surgery. Both chemotherapy and radiation therapy were given to 7.8 % (n = 4) patients.

### Survival analysis

Of the 18 patients with vital status recorded, median follow-up was 45.2 months (IQR 30.3–63.3 months) and overall median survival was 51.1 months (IQR 30.3–63.3 months). 10 patients died of disease with a median follow-up of 34.0 months, while 8 were alive with a median follow-up of 64.9 months. When stratified by therapy, median survival was not reached in patients undergoing surgery alone. Among patients with surgery and chemotherapy alone, only one patient had follow-up data.

Of the three patients who had both surgery and radiation and had follow-up data all three died with a median survival of 51.1 months (IQR 24.5–58.5 months). Median survival of patients undergoing surgery, chemotherapy and radiotherapy was 69.2 months (IQR 20.5–118 months) (Table 2). One patient who received surgery and chemotherapy alone died at 23 months.

**Table 2:** Survival outcomes of National Cancer Database patients stratified by therapy

	Median (IQR)
Median overall survival (months)	51.1 (30.3–63.3)
Median survival (months)	
Surgery alone (n = 10)	
Surgery + chemotherapy (n = 1)	
Surgery + radiotherapy (n = 3)	51.1 (24.5–58.5)
Surgery + chemotherapy + radiotherapy (n = 2)	69.2 (20.5–118)

*IQR* inter-quartile range

### Results of systematic review

The search strategy resulted in 55 articles of which 8 satisfied the inclusion criteria, with a total of 24 cases (Fig. 1; Table 3). 47 articles were excluded from the final analysis because these were published in non-English language, or were single patient case reports or were available as abstracts only. Median age was 47.5 years (IQR 39–66.5 years) and 58.3 % (n = 14) were male (Table 1). Mean tumor size was 12 cm (IQR 7–17 cm). Two of the identified cases were originally diagnosed as hemangiopericytomas while one was identified as deep fibrous histiocytoma. Of 21 cases with CD34 status available, 19 were positive. Similarly 12 cases were bcl-2 positive (of 12 avaialble reports) and 6 CD99 positive (of 7 available reports). 91.7 % (n = 22) patients underwent surgical excision (1 patient had positive surgical margins), while 1 patient also received adjuvant chemotherapy. Of the remaining two patients, only biopsies were performed for one case as multiple comorbidities precluded surgical excision and treatment information was not available in one patient. Median follow up was 54 months (IQR 28–144 months). Late recurrences were reported in 16.7 % (n = 4) cases (2 local and 2 distant recurrences). Recurrence free survival (RFS) ranged from 1 to 288 months. At last follow-up date, 79.2 % (n = 19) patients were alive without disease, 4.2 % (n = 1) alive with disease, 12.5 % (n = 3) died of the disease and one patient was lost to follow-up. Two of the deaths had late recurrences and one died within 30 days of surgery with a positive surgical margin.

**Table 3:** Summary of studies selected for literature review

Authors	Year	N	Average tumor size (cm)	Median follow-up (months)	Time to recurrence (months)
Decouvelaere et al. [9]	1998	3	10.5	120	12–168
Hasegawa et al. [3]	1999	8	10.25	57	
Morimitsu et al. [36]	2000	1	23	32	
Guillou et al. [37]	2000	3	18.17	39^b^	
Clayton et al. [35]^a^	2001	2	10	36	
Takizawa et al. [6]	2007	4	15.9		
Mosquera et al. [17]	2009	1	20	1^c^	
Baldi et al. [2]	2013	2		228	156–276

^a^Adjuvant chemotherapy was given to one of the patients

^b^One patient was lost to follow-up

^c^Patient died within 30 days of surgery

## Discussion

In our study, we analyzed 51 cases of retroperitoneal SFTs from the National Cancer Database on their treatment modality and outcomes and compared it to published literature on retroperitoneal SFTs. To the authors’ best knowledge, this is the largest and most comprehensive series on retroperitoneal SFTs till date and the first study to conduct a formal analysis of surgical outcomes. Based on our study, surgery is a valid standard therapy for retroperitoneal SFTs.

Peri-operative morbidity and mortality rates were comparable with other retroperitoneal sarcomas [15, 16]. When stratified by therapy median survival of patients who underwent surgery and chemotherapy and radiation therapy was higher than patients with surgery and radiotherapy alone but it carries no statistical significance as the former group consisted of four patients of which follow-up information was available only for two. The efficacy of chemotherapy in retroperitoneal SFTs is questionable. When compared to patients who did not receive chemotherapy, chemotherapy group had a larger mean tumor size (18.2 cm vs. 15.8 cm), higher grade (40 % poorly differentiated/undifferentiated vs. 17 %) and more positive resection margins (20 vs. 17 %) and this might have confounded the results.

Retroperitoneal SFTs are different from other SFTs in their larger size at presentation. Retroperitoneal SFTs are mostly asymptomatic or present with vague non-specific symptoms of abdominal pain, increase in abdominal girth, weight loss and are often diagnosed incidentally when imaging for unrelated conditions, while SFTs of pleura, extremities and other sites present with signs and symptoms of mass lesion or are visible to the patient and is likely to be diagnosed early in their course [17]. Thus, the larger size at presentation could be explained by the delay in diagnosis due to lack of specific symptoms. Previous studies have reported a larger tumor size (>10–15 cm) as a predictor of worse outcome for metastasis [10, 18, 19]. Also, size greater than 10 cm is a component of England criteria for classifying SFTs into malignant and benign [5].

Management of retroperitoneal SFTs is not clearly defined. Data is lacking on treatment strategies owing to the orphan status of the tumor. Current therapy is drawn from data on therapy outcomes in similar tumors. Surgery is the standard treatment modality for retroperitoneal SFTs. A positive resection margin has been correlated with worse local recurrence free survival and metastasis free survival by Gold et al. and with local recurrence by van Houdt et al. [10, 19]. However, Wilky et al. has found no association of resection margins with recurrence and instead reported an association with malignant histology suggesting that histology may have confounded the association [20]. Regardless, the authors feel that it is safe to keep a high index of suspicion for recurrence in any tumor with positive resection margins as all the deaths due to disease in the systematic review had positive resection margins while all of the alive patients had negative margins. Recurrence rates in retroperitoneal SFTs are low compared to other retroperitoneal sarcomas (17 vs. 52–61 %) [15, 16]. This might argue against routine use of therapies to reduce recurrence rate unless validated by further evidence. However, the use of radiation therapy in the treatment of extra-abdominal SFT has been documented in case series with anecdotal effectiveness [2, 10].

The rates of peri-operative morbidity and mortality in NCDB data was minimal. Wignall et al. has reported technical complexity of the surgery due to the vascular nature of the tumor and the presence of collateral feeding vessels and recommends referral to a tertiary care center and use of techniques like embolization before surgery [21].

Solitary fibrous tumors may recur even after a prolonged latent period and retroperitoneal SFTs are no exception, as three of the recurrences in the review occurred after 10 years of surgery for primary tumor, as echoed by previous reports [9, 10, 18]. Furthermore, intra-abdominal and retroperitoneal SFTs have been reported to have a higher recurrence rate compared to extra-abdominal tumors [11]. These observations warrant a prolonged follow-up and surveillance for retroperitoneal SFTs. The authors recommend a minimum follow-up of 15 years with prolonged follow-up intervals after first 3 years.

The diagnosis of retroperitoneal SFTs requires histologic confirmation. The lack of a uniform nomenclature system in the past had led to SFTs being identified as localized fibrous tumor, localized fibrous mesothelioma, solitary fibrous mesothelioma, fibrous mesothelioma, subserosal fibroma and submesothelial fibroma [22]. Also, the absence of sensitive and specific markers and the nonspecific histologic pattern of the tumor prohibited an accurate diagnosis. However recent investigations into the genetics of the tumor has revealed the overexpression of STAT6 protein, a result of gene-fusion on chromosome 12q13 which leads to a NAB2-STAT6 fusion product. A highly sensitive and specific marker for SFTs has been developed based on a nuclear staining test for the STAT6 protein solving the diagnostic conundrum [23–26]. SFTs can be divided into benign and malignant categories based on its histological features, namely hyper cellularity, pleomorphism, necrosis/hemorrhage and mitoses (>4 mf/10 hpf). Different criteria have been developed to assign malignant or benign classification to SFTs based on histologic features, age, location, size, sessile/pedunculated nature [5, 18]. Nevertheless, SFTs classified as benign have been observed to undergo malignant transformation and recur at local and distant sites [8, 9, 12, 17, 27]. In view of this unpredictable behavior and the increased risk of retroperitoneal SFTs to recur than other SFTs, it is judicious to follow-up all cases of retroperitoneal SFTs irrespective of satisfying the criteria for benign tumors.

The treatment of recurrent SFTs is evolving. The uncommonness of the tumor prohibits a prospective trial. Anti-angiogenic drugs have been tried considering the vascular nature of the tumor. Interferon-alfa and bevacizumab–temozolomide combination has shown promise in disease stabilization in recurrent cases [28–30]. Tyrosine kinase inhibitors(TKI)s are another set of drugs being investigated for malignant and aggressive SFTs. Sunitinib, pazopanib and regorafenib has been shown to be efficacious in animal models and has been studied retrospectively [31–33]. Conventional chemotherapeutic agents like dacarbazine also has been shown to be effective against SFTs [28, 33]. Prospective studies on dacarbazine, TKIs and other investigational drugs are being conducted currently. Park et al. recommends targeted therapies/antiangiogenic drugs initially and stabilizing the disease later with conventional chemotherapy [34].

Given the rarity of solitary fibrous tumors of the retroperitoneum, studies that describe the management and outcomes of these tumors are limited in number. Available literature is focused on SFTs in the pleura due to its more common occurrence. Previous studies on retroperitoneal SFTs are mostly case reports and outcomes of surgery have not been studied.

Our study was limited by the number of cases, both in the NCDB registry and published literature. Number of patients was insufficient to perform a multi-variate subgroup analysis. Past studies on SFTs were heterogeneous in their reporting of surgeries. Treatment of recurrences was not recorded in some instances. The ambiguous pathologic terminology in practice for describing SFTs makes it possible that many tumors were misclassified and as a result not included in the literature review as our search strategy did not consider all available terms for SFTs. Three of the studies in the systematic review were from the United States and one of them is from a National Cancer Institute designated cancer center and this may lead to an overlap of two patients with the NCDB data [35].

## Conclusions

Considering the orphan status of solitary fibrous tumors, it is impossible to conduct a study involving adequate number of cases. In this scenario, our analysis of NCDB and systematic review of published literature demonstrates that surgical excision is a feasible and reasonable first line of therapy for retroperitoneal solitary fibrous tumors with minimal perioperative morbidity and mortality and overall median survival above 4 years. The diagnosis may be confirmed by novel nuclear staining techniques if in doubt. For recurrent and aggressive tumors, a multimodality therapy incorporating surgery, chemotherapy and radiotherapy may be considered on a case to case basis. Anti–angiogenic drugs and TKIs are appropriate for initial therapy and conventional chemotherapy may be used later to stabilize the disease and the patient could be enrolled in ongoing clinical trials. Given the propensity for late recurrences even in clinically and histologically benign tumors, SFTs should be followed up for a prolonged period of 15 years, irrespective of nature of the tumor at initial presentation.
